# Dysregulation of microRNA biogenesis in cancer: the impact of mutant p53 on Drosha complex activity

**DOI:** 10.1186/s13046-016-0319-x

**Published:** 2016-03-12

**Authors:** Aymone Gurtner, Emmanuela Falcone, Francesca Garibaldi, Giulia Piaggio

**Affiliations:** Department of Research, Advanced Diagnostics, and Technological Innovation, Regina Elena National Cancer Institute, 00144 Rome, Italy

**Keywords:** mutp53, miRNA, Drosha, p68, p72, Biogenesis, Microprocessor complex

## Abstract

A widespread decrease of mature microRNAs is often observed in human malignancies giving them potential to act as tumor suppressors. Thus, microRNAs may be potential targets for cancer therapy. The global miRNA deregulation is often the result of defects in the miRNA biogenesis pathway, such as genomic mutation or aberrant expression/localization of enzymes and cofactors responsible of miRNA maturation. Alterations in the miRNA biogenesis machinery impact on the establishment and development of cancer programs. Accumulation of pri-microRNAs and corresponding depletion of mature microRNAs occurs in human cancers compared to normal tissues, strongly indicating an impairment of crucial steps in microRNA biogenesis. In agreement, inhibition of microRNA biogenesis, by depletion of Dicer1 and Drosha, tends to enhance tumorigenesis in vivo. The p53 tumor suppressor gene, TP53, is mutated in half of human tumors resulting in an oncogene with Gain-Of-Function activities. In this review we discuss recent studies that have underlined a role of mutant p53 (mutp53) on the global regulation of miRNA biogenesis in cancer. In particular we describe how a new transcriptionally independent function of mutant p53 in miRNA maturation, through a mechanism by which this oncogene is able to interfere with the Drosha processing machinery, generally inhibits miRNA processing in cancer and consequently impacts on carcinogenesis.

## Background

**Fig. 1:**
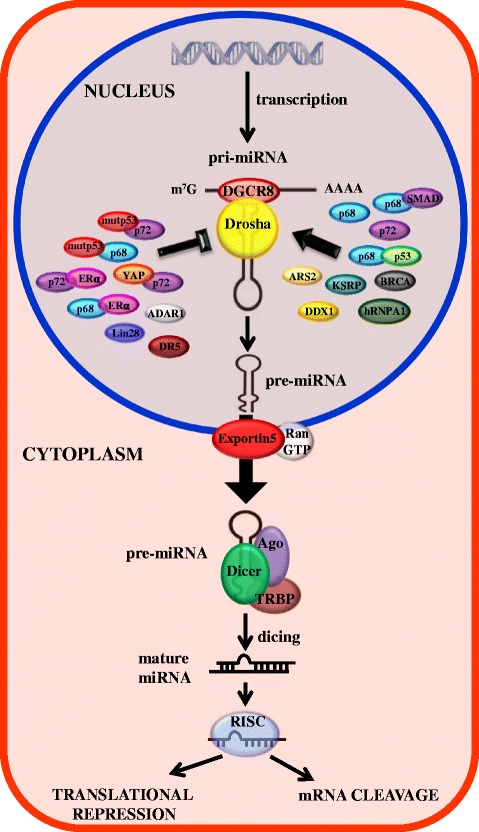
Schematic representation of canonical miRNA biogenesis pathway. The miRNA biogenesis is a multistep process. miRNA genes are transcribed by polymerase II or III resulting in primary precursors (pri-miRNAs). Second, pri-miRNAs are cleaved into pre-miRNA by the Microprocessor complex (Drosha-DGCR8) in the nucleus and then transported from the nucleus to the cytoplasm by Exportin-5-Ran-GTP. Drosha/DGCR8 is subjected to complex regulation by positive and negative factors, represented on the figure. In the cytoplasm, Dicer1, TRBP and Paz protein cleave and digest the pre-miRNA to produce a mature duplex miRNA. The functional strand of the mature miRNA is loaded together with AGO-2 proteins into the RISC. The mature miRNA silences target mRNAs through mRNA cleavage or translational repression

MicroRNAs (miRNAs) are small non coding single-stranded RNAs of about 20–25 nucleotides in length that regulate gene expression by binding to complementary target mRNAs and promoting their decay or inhibiting their translation [1–3]. 1881 human mirRNA loci, annotated on miRBase21, and an even greater number of predicted miRNA targets have been identified in the human genome. Thus, miRNAs are potent regulators of gene expression involved in diverse physiological processes, such as normal development, differentiation, growth control, apoptosis, and in human diseases, particularly in cancer where they act as regulators of key cancer-related pathways [4–6]. The expression level of biologically active mature miRNAs is the result of a fine mechanism of biogenesis, carried out by different enzymatic complexes that exert their function at transcriptional and post-transcriptional levels. MiRNAs sequences are distributed all throughout the genome, being localized in exonic or intronic regions, as well as intergenic locations [7]. The biogenesis of miRNAs starts with their transcription by RNA polymerase II, [8] although some other miRNAs are transcribed by RNA polymerase III, [9] resulting in a primary transcript known as pri-miRNA which have a stem-loop structure, are capped at the 5′-end and have a 3′-poly (A) tail. The canonical miRNA biogenesis pathway is characterized by two subsequent central steps utilizing ribonuclease reactions (Fig. 1). In the nucleus, pri-miRNAs are recognized and cropped into hairpin-structured precursor miRNAs (pre-miRNAs) by the Drosha complex (also known as Microprocessor complex). Drosha, an RNase III enzyme, and DGCR8 (DiGeorge critical region 8), a double-stranded RNA-binding domain (dsRBD) protein, are two essential components of the Microprocessor complex. Drosha liberates the stem_loop pre-miRNAs from pri-miRNAs in cooperation with DGCR8-mediated recognition of the junctional region between the single-stranded and double-stranded portions of pri-miRNAs [10, 11]. The Drosha complex also contains several auxiliary factors including the DEAD-box RNA helicases p68 (DDX5) and p72/p82 (DDX17) which promote the fidelity and activity of Drosha processing [12]. The pre-miRNAs of approximately 70 nucleotides in length are transported from the nucleus to the cytoplasm by Exportin-5 (XPO5) together with Ran-GTP [13]. In the cytoplasm, Dicer, another RNase III, digests the pre-miRNA into a 20–25 nucleotides mature duplex miRNA. During this process, Dicer is associated with other proteins like TAR RNA binding protein (TRBP) and kinase R–activating protein (PACT) to increase its stability and its processing activity [14, 15]. The miRNA duplex is comprised of two miRNA strands, with one strand loaded onto the RNA-induced silencing complex (RISC), which contains the Argonaute (Ago) family protein as a core component. In these processes, the other strand (miRNA* strand) is usually degraded, however, in some cases miRNA* strands are retained and function [16]. Mature miRNAs serve as guides directing RISC to target mRNAs, which are degraded, destabilized or translationally inhibited by the Ago proteins [17].

Deregulation of miRNAs is commonly observed in human cancers [17]. Microarray expression data from a wide spectrum of cancer diseases have evidenced a general aberrant miRNA expression in cancer [18–20] and a large amount of data have revealed that the physiological state of cancer cells can be detected by miRNA expression profiling [21, 22]. Importantly, single miRNA may target up to several hundred mRNAs, indeed an aberrant miRNA expression may profoundly influence cancer-related signaling pathways. In agreement, mouse models displaying miRNA overexpression or depletion have demonstrated causal links between miRNAs and cancer development making them putative therapeutic targets [22, 23]. Although miRNAs can function as both tumour suppressors and oncogenes in tumour development, a widespread down-regulation of miRNAs is commonly observed in human cancers [5, 17, 24, 25], suggesting that miRNAs are primarily tumor suppressor genes. The global down-regulation in miRNA expression arises through multiple mechanisms: genetic alterations [26–28], transcriptional regulation (epigenetic mechanisms, miRNA suppression by oncogenic transcription factors, miRNA downregulation by loss of tumour suppressor transcription factors) and post-transcriptional regulation [29, 30]. An increasing number of evidences indicate that post-transcriptional maturation, rather than transcription, is often perturbed in cancer. The accumulation of pri-miRNAs and the corresponding depletion of mature miRNAs have been evidenced in human cancers compared to normal tissue [31], strongly indicating that the impairment of crucial steps in miRNA biogenesis could be the underlying cause.

Recent studies have identified that deregulated expression and subcellular localization of Drosha, Dicer and TARBP1 correlate with disease progression and poor prognosis in different types of tumors [30]. Moreover, genetic defects in genes encoding Dicer1 [32], exportin5 [33] and TARBP2 [34] have been found underscoring the relevance of the processing machinery of miRNA in cancer. Recently two studies showed that mutations in the SIX1/2 pathway and the DROSHA/DGCR8 miRNA Microprocessor complex underlie high-risk blastemal type Wilms tumors [35, 36]. Finally the repression of miRNA processing by the partial depletion of Dicer1 and Drosha accelerates cellular transformation and tumorigenesis in vivo [37].

The biogenesis of miRNAs requires not only the core processing enzymatic machinery, but also many regulatory factors, such as RNA binding factor or transcription factors often deregulated in cancer cells. A panel of modulators that have been reported to influence the processing of pri-miRNAs including p68 (DDX5), p72/p82 (DDX17), DDX1, BRCA, ARS2, DR5, ADAR1, hRNP A1, KSRP, Lin 28, SMADs, YAP, ERα, ERβ, wtp53 and mutant p53 [38–58] (Fig. 1), imply that there is a link between oncogene and oncosuppressors pathways and the Microprocessor complex.

p68 (DDX5) and p72/p82 (DDX17) are prototypic members of the DEAD box protein family of RNA helicases. Both p68 and p72 are responsible for the processing of a subset of pri-miRNAs [38, 59] and acting as a bridge between Drosha and other proteins can impact on cancer development by regulating known oncogenes and tumor suppressors. Indeed, several molecules, involved in different signalling pathways, have been described to bind p68 and/or p72/82 regulating Drosha/DGCR8 mediated miRNAs processing such as wtp53 and mutp53 [52, 56, 57].

The TP53 tumour suppressor is one of the most important and well-studied cancer gene, and it is not surprising that it has been found to have a role in miRNA regulation. More than 50 % of human cancers carry mutations within the p53 locus [60]. The common types of cancer–associated p53 mutations are missense mutations mainly located in the DNA binding domain of p53. The resulting full-length mutant p53 protein is unable to activate transcription of wtp53 target genes connected with cell cycle arrest, apoptosis and DNA repair [61–63]. Furthermore, mutant p53 (mutp53) proteins can acquire novel oncogenic functions known as gain of function activities (GOF) favouring in vivo tumor induction, maintenance and spreading in mouse models [64]. At molecular level, GOF mutant p53 proteins can exert their activities either through the binding, the sequestration, and the inactivation of tumor suppressor proteins or through the transcriptional regulation of target genes [65, 66]. The widespread nature of p53 mutations in cancer has suggested a relationship between mutp53 GOF activities and the deregulation of miRNA biogenesis observed in cancer. Indeed, the understanding of these mechanisms is a cardinal question in cancer biology. Some very recent experimental efforts have provided substantial advances in understanding miRNA biogenesis and its regulation by p53 proteins. These findings will impact on the knowledge of the molecular mechanisms through which mutp53s exert their GOF activities, and regulate gene expression, by adding to this puzzle the ability to interfere with miRNA processing.

## Wtp53-Microprocessor complex interplay

The first evidence of a direct involvement of wtp53 on miRNA biogenesis has been described by Suzuki and collaborators in 2009 [52]. They found that in colon carcinoma cell line HCT116, after DNA damage (Doxorubicine), wtp53 interacts with the Drosha complex through its DNA binding domain, facilitating the processing of a subset of nine pri-miRNAs including pri-miR-16-1, −143, −145 and −206, with growth suppressive function. They found that wtp53 expression induced an increase in the p68 association to Drosha–complex that in turn increases Drosha activity. Moreover, their data strongly indicate that p53 interacts with the Drosha complex through RNA molecules and p68. The authors also demonstrated that several of the wtp53-regulated miRNAs decrease cell proliferation rate targeting k-Ras (miR-143), and CDK6 (miR-16, 26a, −107, −145, −206). These results underlie a novel function of wtp53 in miRNA maturation and strongly indicate a role for wtp53 controlling global gene expression and cell fate by positively modulating Microprocessor activity. Subsequently three different groups have described a role of the wtp53 on pri-miRNA processing. In a recent work Kazuya Nakazawa and colleagues provide evidences regarding the role of an activated p53 on pri-miR-1915 processing in response to DNA damage (Adriamicine) [53]. J Chang and colleagues found that after HCT116 cells treatment with the DNA damaging agent camptotecina (CPT), that induces wtp53 accumulation and consequently apoptosis, wtp53 leads to the expression of 5 miRNAs (miR-16, −103, −143, −203, 206) at post-transcriptional level [54]. Of note the authors found a mechanism by which the acetylation of K120 in the DNA-binding domain of wtp53, which play a critical role in mediating apoptosis, augments its association with the Drosha Microprocessor complex and the interaction between Drosha and p68. In agreement, the knockdown of hMOF, the acetyltransferase that targets K120 in p53, strongly reduces miRNA induction and apoptosis mediated by CPT. Finally the authors found that downregulation of anti-apoptotic Bcl-w by miR-203 is necessary for cell death upon CPT treatment indicating that post-transcriptional regulation of gene expression by wtp53 via miRNAs plays a role in determining stress-specific cellular outcomes. Moreover, altogether these findings strongly suggest that the mutated p53^K120R^ found in tumors may contribute to tumorigenesis and to resistance to chemotherapy also through deregulation of miRNA biosynthesis. Of interest, other post translational modifications that are induced after DNA damage and play a key role on cellular response, globally induce miRNA biogenesis. Indeed, after DNA damage, ATM kinase directly binds to and phosphorylates KSRP, leading to enhanced interaction between KSRP and pri-miRNAs increasing miRNA processing by Drosha [67]. Two papers underline a SIRT1-wtp53-miR34a axis. Herbert KT et al. have described that SIRT1 modulates wtp53 acetylation statusthus promoting maturation of pri-miRNAs transcripts, among which miR-34a [55].

A SIRT1-wtp53-miR34a axis has been also described in which SIRT1, de-acetylating wtp53 (K382), inhibits pri-miR-34a maturation in normal keratinocytes. Interestingly, this SIRT1 dependent regulation of miRNA biogenesis seems to be impaired on keratinocyte cell line with p53 mutations. Nevertheless, by the experiments described on the manuscript it is not clear which step of miRNA biogenesis is governed by wtp53-SIRT cross-talk [68]. Interestingly, wtp53 induces a RNA-binding protein, RBM38, that blocks miRNAs accessibility to 3′UTR of several wtp53 target genes that promote cell death upon genotoxic stress, such as the 3′UTR of p21- [69]. Notably the accessibility of the 3′UTR of SIRT1, that is targeted by miR-34a promoting cell survival, is not under RBM38 regulation.

## Impact of mutp53 on miRNA biogenesis

In 2009, in the same manuscript concerning the role of wtp53 in miRNA processing, Suzuki and collaborators provide evidences that the overexpression of three tumor GOF-p53 mutants in the DNA binding domain (R273H, R175H, C135Y) in colon carcinoma HCT p53-null cell line, decreases the ability of an ectopic expressed Drosha to bind p68 and pri-miRNAs, leading to inhibition of processing of three wtp53-dependent-miRNAs [52]. The last was the first experimental data suggesting the idea that a novel oncogenic property of mutp53 is the attenuation of miRNA post-transcriptional maturation.

Later, in the 2015, Jiang FZ et al. found that the overexpression of different mutp53 proteins (C135Y, R175H, R248Q, R273H) in p53-null endometrial carcinoma cell lines (HEC-50) disrupts p68-Drosha complex assembly and inhibits the recruitment of Drosha on pri-miRNA-26a1 [56], confirming in part the results obtained by Suzuki on colon cancer cells [52]. Interestingly, the authors demonstrated that in HEC-1B cells, endogenously expressing mutantp53-R248Q, the last binds p68 suggesting that it titrates this RNA helicase from the Microprocessor complex. In these cells, mutp53 promotes the epithelial to mesenchymal transition by repressing miR-26a expression that in turn inhibits EZH2 (the transcriptional repressor Enhancer of Zeste Homolog2), both in vitro and in xenograft experiments [56]. EZH2 is an oncogene upregulated in several tumors that epigenetically silences a plethora of oncosuppressor genes [70]. Thus, due to the widespread activity of EZH2 on gene expression regulation, the biological effects of mutant p53-dependent regulation of miRNAs that modulate this oncogene, would profoundly impact on cell cancer biology.

Very recently, we have demonstrated that the endogenous GOF-mutp53 proteins modulate the biogenesis of several miRNAs in cancer cells directly interfering with Drosha-p72 association and promoting cell survival and cell migration [57]. By a genome wide analysis of miRNA expression on colon cancer cell line SW480, we observed that, upon endogenous mutp53-R273H depletion, frequently present in human tumors, 33 out of 376 miRNAs analyzed were upregulated and only four downregulated, strongly indicating, for the first time, that mutp53 may be responsible at least in part for the miRNAs downregulation observed in cancer. Moreover, we found that another missense mutp53-R175H, inhibits the expression of several of these miRNAs in breast cancer cells (SKBR3). This strongly points out a general mechanism that involves different p53 proteins with missense mutations and suggests that the signature of miRNAs downregulated by mutp53 proteins in different solid tumors has, at least in part, common members. From a mechanistic point of view, through pri- pre- and mature form analysis, we found that mutp53 downregulates miRNAs not only at transcriptional but also at post-transcriptional level. In agreement, we demonstrated that endogenous mutp53 proteins (R273H and R175H) directly bind p72/82 through its N-terminal domain, hindering the association of this DEAD-box with the Microprocessor complex and pri-miRNAs, and leading to the inhibition of the biogenesis of a subset of miRNAs positively regulated by p72. Of note we found that the endogenous wtp53 has an opposite effect on the expression of mutp53 repressed miRNAs on colon cancer cell lines confirming the contribution of mutant p53 GOF on miRNA repression.

On the other hand, in 2014, Li and colleagues, as well, have provided evidences that mutp53 can promote microRNA biogenesis [58]. The authors demonstrate that different mutp53 regulate the amount of ERα protein by regulating the biogenesis of miR-18a, which in turn decreases the tumor-protective function of the estrogen pathway in female hepatocarcinogenesis [58, 71]. Although the authors don’t describe in detail the molecular mechanism, they provided some evidences of the impairment of pri-miRNA processing driving by mutp53, in cells and in human hepatocellular carcinoma (HCC) samples. They found that the precursor and mature form of miRNA-18a, but not the primary transcript, are upregulated in liver tissues collected from 77 female HCC patients. They identify mutp53 as a putative factor involved in the regulation of the processing of miR-18a, but not of the other member of the oncogenic cluster miR-17 ∼ 92 [72–74]. Moreover, the overexpression of several p53 proteins mutated in the DNA binding domain (K132E, I162F, I232F, R249S), frequently observed in HCC, greatly increase the level of miR-18a and decrease, though at different extent, the levels of its primary transcript. To summarize the data available up today, in Table 1 is shown a list of miRNAs that have been demonstrated to be regulated by mutp53 both a transcriptional and posttranscriptional level. In the same table are also indicated the available data about the biological function of these miRNAs (75-93).

**Table 1:** MiRNAs regulated by mutant p53

miRNA	References	Biological functions	References
Transcriptionally regulated			
27a	75	Cell growth, tumorigenesis	75
let7i	76	Inhibition of cell invasion and migration	76
130b	77	EMT repression	77
155	78	Cell invasion, metastasis	78
128-2	79	Anti-apoptotic, chemoresistance	79
223	80	Pro-apoptotic	80
520 g	57	Chemoresistance	81
518b	57	Inhibition of cell proliferation and invasion	82
582	57	Inhibition of cell proliferation and invasion	83
141	57	EMT repression	84
519c	57	Inhibition of angiogenesis	85
143	57	cell cycle arrest, apoptosis	86, 87
142	57	Inhibition of cell proliferation, migration, invasion	88
Post-transcriptionally regulated			
26a	56	Repression of EMT and invasion	56
16	52	Inhibition of cell growth and angiogenesis	52
206	52	Inhibition of cell growth and angiogenesis	93
18a	58	Cell proliferation, invasion, tumorigenesis	58
517a	57	Inhibition of proliferation, apoptosis	57, 89
218	57	Inhibition of EMT and invasion	57, 90
519a	57	Inhibition of proliferation, migration and EMT	57, 91
105	57	Inhibition of proliferation	57
628	57	Unknown	
515	57	Inhibition of proliferation, apoptosis	92
1	57	Suppression of cell growth, invasion, metastasis	93

The table lists miRNAs regulated by mutp53 both at transcriptional and post-transcriptional level. Biological functions of miRNAs and references are mentioned (on the left references relatives to the mut p53-dependent regulation, on the right those referred to their biological role)

## Conclusions

Collectively in the last five years, several experimental evidences in different cellular contexts have demonstrated that several GOF-mutp53 regulate miRNA biogenesis by acting on Drosha complex activity.

**Fig. 2:**
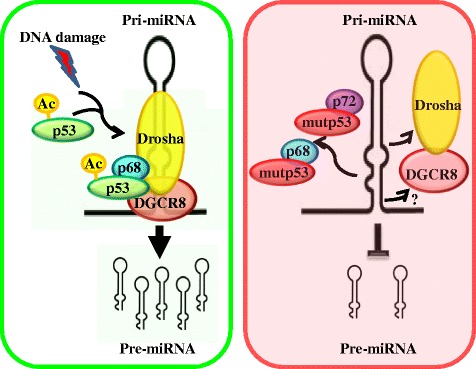
Representation of molecular mechanisms through which wtp53 (*green*) and mutp53 (*red*) impact on Drosha activity. Upon DNA damage, the complex p53/p68 binds the microprocessor complex fostering the maturation of a subset of pri-miRNAs to pre-miRNAs. In a cellular context in which mutp53 is expressed, the last binds p68 and p72, the Microprocessor complex is dissociated and the maturation of a subset of pri-miRNAs to pre-miRNAs is impaired

**Fig. 3:**
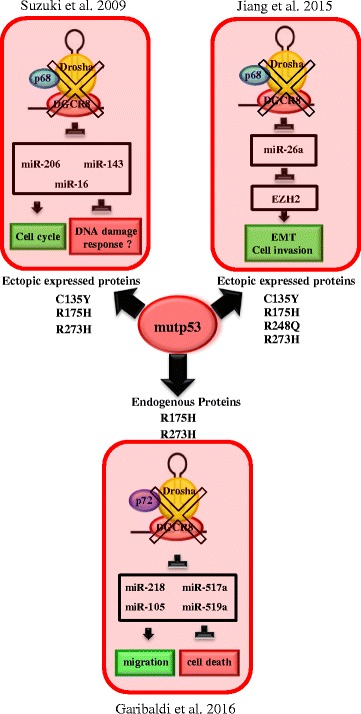
pri-miRNA regulation by mutp53 proteins and functions. In the figure the three published papers providing demonstrations of the impact of mutp53 isoforms on Microprocessor complex are highlighted. The miRNAs described in the papers and the functional consequences of their downregulation are also indicated

Based on the evidences available so far, two molecular mechanisms underlying the mutp53-dependent modulation of miRNA processing can be proposed: 1) wtp53 increases pri-miRNA biogenesis by a direct binding with Drosha and p68 cofactors, while, on the contrary, mutp53 suppresses miRNA processing activity of Drosha by sequestering p68; 2) mutp53 directly binds p72/82 and represses Drosha activity (Fig. 2). By a functional point of view, these two molecular mechanisms lead to an increase of cell migration, epithelial to mesenchymal transition and cell survival (Fig. 3).

p68 and p72/82, as co-factor of the Microprocessor complex, are involved in the Drosha miRNA processing of several but not all miRNAs. Masaki Mori and collaborators [49] have provided some findings suggesting that p72 selectively binds a VCAUCH sequence motif present in the 3′ of some pri-miRNAs to enhance the Drosha processing. Although, the precise role of these two RNA helicases in pri-miRNA processing is unknown. Further biochemical and bioinformatics experiments are needed to better identify the sequence motif and structures of pri-miRNAs that are selectively regulated by the mutp53/p72/Drosha and mutp53/p68/Drosha axis.

Since mutations in TP53 occur at high frequency in human cancers, developing strategies to block the oncogenic effects of mutp53 will be an important step for their treatment. By this point of view, it is important to notice that, mutant p53, as well as wtp53, impact on miRNA biogenesis not only at the level of Drosha but also regulating miRNA transcription as well as maturation from pre- to mature miRNAs. Thus, since miRNAs are powerful regulators of gene expression, the biological effects of miRNAs downregulation by mutp53 are profound.

It would be interesting in the future to identify, on a larger scale, the entire repertoire of miRNAs negatively downregulated by different mutp53 in different tumor models and in human samples. The characterization of the entire gene-regulatory networks governed by mutp53-miRNA cross-talks will offer a molecular basis for diagnostic and therapeutic strategies based on miRNA biology. Indeed, it is possible to envisage that a specific miRNA signature, both in human tissues or in biological fluids, such as plasma, could be useful to predict mutant p53 status.

## References

[1] Ha M, Kim VN (2014). Regulation of microRNA biogenesis. Nat Rev Mol Cell Biol.

[2] Krol J, Loedige I, Filipowicz W (2010). The widespread regulation of microRNA biogenesis, function and decay. Nat Rev Genet.

[3] Winter J, Jung S, Keller S, Gregory RI, Diederichs S (2009). Many roads to maturity: microRNA biogenesis pathways and their regulation. Nat Cell Biol.

[4] Bushati N, Cohen SM (2007). microRNA functions. Annu Rev Cell Dev Biol.

[5] Lujambio A, Lowe SW (2012). The microcosmos of cancer. Nature.

[6] Lynam-Lennon N, Maher SG, Reynolds JV (2009). The roles of microRNA in cancer and apoptosis. Biol Rev Camb Philos Soc.

[7] Altuvia Y, Landgraf P, Lithwick G, Elefant N, Pfeffer S, Aravin A (2005). Clustering and conservation patterns of human microRNAs. Nucleic Acids Res.

[8] Lee Y, Kim M, Han J, Yeom KH, Lee S, Baek SH (2004). MicroRNA genes are transcribed by RNA polymerase II. EMBO J.

[9] Borchert GM, Lanier W, Davidson BL (2006). RNA polymerase III transcribes human microRNAs. Nat Struct Mol Biol.

[10] Lee Y, Ahn C, Han J, Choi H, Kim J, Yim J (2003). The nuclear RNase III Drosha initiates microRNA processing. Nature.

[11] Han J, Lee Y, Yeom KH, Nam JW, Heo I, Rhee JK (2006). Molecular basis for the recognition of primary microRNAs by the Drosha-DGCR8 complex. Cell.

[12] Gregory RI, Yan KP, Amuthan G, Chendrimada T, Doratotaj B, Cooch N (2004). The Microprocessor complex mediates the genesis of microRNAs. Nature.

[13] Zeng Y, Cullen BR (2004). Structural requirements for premicroRNA binding and nuclear export by Exportin 5. Nucleic Acids Res.

[14] Lee Y, Hur I, Park SY, Kim YK, Suh MR, Kim VN (2006). The role of PACT in the RNA silencing pathway. EMBO J.

[15] Chiang HR, Schoenfeld LW, Ruby JG, Auyeung VC, Spies N, Baek D (2010). Mammalian microRNAs: experimental evaluation of novel and previously annotated genes. Genes Dev.

[16] Bartel DP (2009). MicroRNAs: target recognition and regulatory functions. Cell.

[17] Jansson MD, Lund AH (2012). MicroRNA and cancer. Mol Oncol.

[18] Croce CM (2009). Causes and consequences of microRNA dysregulation in cancer. Nat Rev Genet.

[19] Lu J, Getz G (2005). MicroRNA expression profiles classify human cancers. Nature.

[20] Munker R, Calin GA (2011). MicroRNA profiling in cancer. Clin Sci (Lond).

[21] Yanaihara N, Caplen N, Bowman E, Seike M, Kumamoto K, Yi M (2006). Unique microRNA molecular profiles in lung cancer diagnosis and prognosis. Cancer Cell.

[22] Calin GA, Ferracin M, Cimmino A, Di Leva G, Shimizu M, Wojcik SE (2005). A microRNA signature associated with prognosis and progression in chronic lymphocytic leukemia. N Engl J Med.

[23] Iorio MV, Croce CM (2012). MicroRNA dysregulation in cancer: diagnostics, monitoring and therapeutics. A comprehensive review. EMBO Mol Med.

[24] Van Kouwenhove M, Kedde M, Agami R (2011). MicroRNA regulation by RNA-binding proteins and its implications for cancer. Nat Rev Cancer.

[25] Fabbri M, Ivan M, Cimmino A, Negrini M, Calin GA (2007). Regulatory mechanisms of microRNAs involvement in cancer. Expert Opin Biol Ther.

[26] Calin GA, Sevignani C, Dumitru CD, Hyslop T, Noch E, Yendamuri S (2004). Human microRNA genes are frequently located at fragile sites and genomic regions involved in cancers. Proc Natl Acad Sci U S A.

[27] Lagana A, Russo F, Sismeiro C, Giugno R, Pulvirenti A, Ferro A (2010). Variability in the incidence of miRNAs and genes in fragile sites and the role of repeats and CpG islands in the distribution of genetic material. PLoS One.

[28] Mayr C, Hemann MT, Bartel DP (2007). Disrupting the pairing between let-7 and Hmga2 enhances oncogenic transformation. Science.

[29] Romero-Cordoba SL, Salido-Guadarrama I, Rodriguez-Dorantes M, Hidalgo-Miranda A (2014). miRNA biogenesis: biological impact in the development of cancer. Cancer Biol Ther.

[30] Liu X, Chen X, Yu X, Tao Y, Bode AM, Dong Z (2013). Regulation of microRNAs by epigenetics and their interplay involved in cancer. J Exp Clin Cancer Res.

[31] Thomson JM, Newman M, Parker JS, Morin-Kensicki EM, Wright T, Hammond SM (2006). Extensive post-transcriptional regulation of microRNAs and its implications for cancer. Genes Dev.

[32] Hill DA, Ivanovich J, Priest JR, Gurnett CA, Dehner LP, Desruisseau D (2009). DICER1 mutations in familial pleuropulmonary blastoma. Science.

[33] Melo SA, Moutinho C, Ropero S, Calin GA, Rossi S, Spizzo R (2010). A genetic defect in exportin-5 traps precursor microRNAs in the nucleus of cancer cells. Cancer Cell.

[34] Melo SA, Ropero S, Moutinho C, Aaltonen LA, Yamamoto H, Calin GA (2009). A TARBP2 mutation in human cancer impairs microRNA processing and DICER1 function. Nat Genet.

[35] Wegert J, Ishaque N, Vardapour R, Geörg C, Gu Z, Bieg M (2015). Mutations in the SIX1/2 pathway and the DROSHA/DGCR8 miRNA microprocessor complex underlie high-risk blastemal type Wilms tumors. Cancer Cell.

[36] Walz AL, Ooms A, Gadd S, Gerhard DS, Smith MA, Guidry Auvil JM (2015). Recurrent DGCR8, DROSHA, and SIX homeodomain mutations in favorable histology Wilms tumors. Cancer Cell.

[37] Kumar MS, Lu J, Mercer KL, Golub TR, Jacks T (2007). Impaired microRNA processing enhances cellular transformation and tumorigenesis. Nat Genet.

[38] Fuller-Pace FV, Moore HC (2011). RNA helicases p68 and p72: multifunctional proteins with important implications for cancer development. Future Oncol.

[39] Hong S, Noh H, Chen H, Padia R, Pan ZK, Su SB (2013). Signaling by p38 MAPK stimulates nuclear localization of the microprocessor component p68 for processing of selected primary microRNAs. Sci Signaling.

[40] Han C, Liu Y, Wan G, Choi HJ, Zhao L, Ivan C (2014). The RNA-binding protein DDX1 promotes primary microRNA maturation and inhibits ovarian tumor progression. Cell Rep.

[41] Kawai S, Amano A (2012). BRCA1 regulates microRNA biogenesis via the DROSHA microprocessor complex. J Cell Biol.

[42] Gruber JJ, Zatechka DS, Sabin LR, Yong J, Lum JJ, Kong M (2009). Ars2 links the nuclear cap-binding complex to RNA interference and cell proliferation. Cell.

[43] Sabin LR, Zhou R, Gruber JJ, Lukinova N, Bambina S, Berman A (2009). Ars2 regulates both miRNA- and siRNA- dependent silencing and suppresses RNA virus infection in Drosophila. Cell.

[44] Nemlich Y, Greenberg E, Ortenberg R, Besser MJ, Barshack I, Jacob-Hirsch J (2013). MicroRNA-mediated loss of ADAR1 in metastatic melanoma promotes tumor growth. J Clin Invest.

[45] Haselmann V, Kurz A, Bertsch U, Hubner S, Olempska-Muller M, Fritsch J (2014). Nuclear death receptor TRAIL-R2 inhibits maturation of let-7 and promotes proliferation of pancreatic and other tumor cells. Gastroenterology.

[46] Michlewski G, Guil S, Semple CA, Caceres JF (2008). Posttranscriptional regulation of miRNAs harboring conserved terminal loops. Mol Cell.

[47] Heo I, Joo C, Kim YK, Ha M, Yoon MJ, Cho J (2009). TUT4 in concert with Lin28 suppresses microRNA biogenesis through premicroRNA uridylation. Cell.

[48] Davis BN, Hilyard AC, Lagna G, Hata A (2008). SMAD proteins control DROSHA-mediated microRNA maturation. Nature.

[49] Mori M, Triboulet R, Mohseni M, Schlegelmilch K, Shrestha K, Camargo FD (2014). Hippo signaling regulates microprocessor and links cell-density-dependent miRNA biogenesis to cancer. Cell.

[50] Paris O, Ferraro L, Grober OM, Ravo M, De Filippo MR, Giurato G (2012). Direct regulation of microRNA biogenesis and expression by estrogen receptor beta in hormone-responsive breast cancer. Oncogene.

[51] Dai TY, Cao L, Yang ZC, Li YS, Tan L, Ran XZ (2014). P68 RNA helicase as a molecular target for cancer therapy. J Exp Clin Cancer Res.

[52] Suzuki HI, Yamagata K, Sugimoto K, Iwamoto T, Kato S, Miyazono K (2009). Modulation of microRNA processing by p53. Nature.

[53] Nakazawa K, Dashzeveg N, Yoshida K (2014). Tumor suppressor p53 induces miR-1915 processing to inhibit Bcl-2 in the apoptotic response to DNA damage. FEBS J.

[54] Chang J, Davis-Dusenbery BN, Kashima R, Jiang X, Marathe N, Sessa R (2013). Acetylation of p53 stimulates miRNA processing and determines cell survival following genotoxic stress. EMBO J.

[55] Herbert KJ, Cook AL, Snow ET (2014). SIRT1 modulates miRNA processing defects in p53-mutated human keratinocytes. J Dermatol Sci.

[56] Jiang FZ, He YY, Wang HH, Zhang HL, Zhang J, Yan XF, et al. Mutant p53 induces EZH2 expression and promotes epithelial-mesenchymal transition by disrupting p68-Drosha complex assembly and attenuating miR-26a processing. Oncotarget. 2015. doi: 10.18632/oncotarget.6350. 10.18632/oncotarget.6350 PMC4792583 26587974

[57] Garibaldi F, Falcone E, Trisciuoglio D, Colombo T, Lisek K, Walerych D, Del Sal G, Paci P, Bossi G, Piaggio G, Gurtner A. Mutant p53 inhibits miRNA biogenesis by interfering with the Microprocessor complex. Oncogene. *In press*. 10.1038/onc.2016.51 26996669

[58] Li CL, Yeh KH, Liu WH, Chen CL, Chen DS, Chen PJ (2015). Elevated p53 promotes the processing of miR-18a to decrease estrogen receptor-α in female hepatocellular carcinoma. Int J Cancer.

[59] Gregory RI, Yan KP, Amuthan G, Chendrimada T, Doratotaj B, Cooch N (2004). The microprocessor complex mediates the genesis of MicroRNAs. Nature.

[60] Muller PA, Vousden KH (2014). Mutant p53 in cancer: new functions and therapeutic opportunities. Cancer Cell.

[61] Oren M (2003). Decision making by p53: life, death and cancer. Cell Death Differ.

[62] Gurtner A, Starace G, Norelli G, Piaggio G, Sacchi A, Bossi G (2010). Mutant p53-induced up-regulation of mitogen-activated protein kinase kinase 3 contributes to gain of function. J Biol Chem.

[63] Ubertini V, Norelli G, D’Arcangelo D, Gurtner A, Cesareo E, Baldari S (2015). Mutant p53 gains new function in promoting inflammatory signals by repression of the secreted interleukin-1 receptor antagonist. Oncogene.

[64] Donehower LA, Lozano G (2009). 20 years studying p53 functions in genetically engineered mice. Nat Rev Cancer.

[65] Donehower LA (2014). Insights into wild-type and mutant p53 functions provided by genetically engineered mice. Hum Mutat.

[66] Gurtner A, Starace G, Norelli G, Piaggio G, Sacchi A, Bossi G (2010). Mutant p53-induced up-regulation of mitogen-activated protein kinase kinase 3 contributes to gain of function. J Biol Chem.

[67] Zhang X, Wan G, Berger FG, He X, Lu X (2011). The ATM kinase induces microRNA biogenesis in the DNA damage response. Mol Cell.

[68] Yamakuchi M, Lowenstein CJ (2009). MiR-34, SIRT1 and p53: the feedback loop. Cell Cycle.

[69] Léveillé N, Elkon R, Davalos V, Manoharan V, Hollingworth D, Oude Vrielink J (2011). Selective inhibition of microRNA accessibility by RBM38 is required for p53 activity. Nat Commun.

[70] Deb G, Singh AK, Gupta S (2014). EZH2: not EZHY (easy) to deal. Mol Cancer Res.

[71] Deng L, Yang H, Tang J, Lin Z, Yin A, Gao Y (2015). Inhibition of MTA1 by ERα contributes to protection hepatocellular carcinoma from tumor proliferation and metastasis. J Exp Clin Cancer Res.

[72] He L, Thomson JM, Hemann MT, Hernando-Monge E, Mu D, Goodson S (2005). A microRNA polycistron as a potential human oncogene. Nature.

[73] Mu P, Han YC, Betel D, Yao E, Squatrito M, Ogrodowski P (2009). Genetic dissection of the miR-17 ∼ 92 cluster of microRNAs in Myc-induced B-cell lymphomas. Genes Dev.

[74] Feng Y, Liu J, Kang Y, He Y, Liang B, Yang P (2014). miR-19a acts as an oncogenic microRNA and is up-regulated in bladder cancer. J Exp Clin Cancer Res.

[75] Wang W, Cheng B, Miao L, Mei Y, Wu M (2013). Mutant p53-R273H gains new function in sustained activation of EGFR signaling via suppressing miR-27a expression. Cell Death Dis.

[76] Subramanian M, Francis P, Bilke S, Li XL, Hara T, Lu X (2015). A mutant p53/let-7i-axis-regulated gene network drives cell migration, invasion and metastasis. Oncogene.

[77] Dong P, Karaayvaz M, Jia N, Kaneuchi M, Hamada J, Watari H (2013). Mutant p53 gain-of-function induces epithelial-mesenchymal transition through modulation of the miR-130b-ZEB1 axis. Oncogene.

[78] Neilsen PM, Noll JE, Mattiske S, Bracken CP, Gregory PA, Schulz RB, et al. Mutant p53 drives invasion in breast tumors through up-regulation ofmiR-155. Oncogene. 2013;32(24):2992–3000. 10.1038/onc.2012.305 22797073

[79] Donzelli S, Fontemaggi G, Fazi F, Di Agostino S, Padula F, Biagioni F (2012). MicroRNA-128-2 targets the transcriptional repressor E2F5 enhancing mutant p53 gain of function. Cell Death Differ.

[80] Masciarelli S, Fontemaggi G, Di Agostino S, Donzelli S, Carcarino E, Strano S (2014). Gain-of-function mutant p53 downregulates miR-223 contributing to chemoresistance of cultured tumor cells. Oncogene.

[81] Zhang Y, Geng L, Talmon G, Wang J (2015). MicroRNA-520 g confers drug resistance by regulating p21 expression in colorectal cancer. J Biol Chem.

[82] Zhang M, Zhou S, Zhang L, Zhang J, Cai H, Zhu J (2012). miR-518b is down-regulated, and involved in cell proliferation and invasion by targeting Rap1b in esophageal squamous cell carcinoma. FEBS Lett.

[83] Zhang X, Zhang Y, Yang J, Li S, Chen J (2015). Upregulation of miR-582-5p inhibits cell proliferation, cell cycle progression and invasion by targeting Rab27a in human colorectal carcinoma. Cancer Gene Ther.

[84] Feng X, Wang Z, Fillmore R, Xi Y (2014). MiR-200, a new star miRNA in human cancer. Cancer Lett.

[85] Cha ST, Chen PS, Johansson G, Chu CY, Wang MY, Jeng YM (2010). MicroRNA-519c suppresses hypoxia-inducible factor-1alpha expression and tumor angiogenesis. Cancer Res.

[86] Borralho PM, Simões AE, Gomes SE, Lima RT, Carvalho T (2011). miR-143 overexpression impairs growth of human colon carcinoma xenografts in mice with induction of apoptosis and inhibition of proliferation. PLoS One.

[87] Zhang J, Sun Q, Zhang Z, Ge S, Han ZG, Chen WT (2013). Loss of microRNA-143/145 disturbs cellular growth and apoptosis of human epithelial cancers by impairing the MDM2-p53 feedback loop. Oncogene.

[88] Wu L, Cai C, Wang X, Liu M, Li X, Tang H (2011). MicroRNA-142-3p, a new regulator of RAC1, suppresses the migration and invasion of hepatocellular carcinoma cells. FEBS Lett.

[89] Liu RF, Xu X, Huang J, Fei QL, Chen F, Li YD (2013). Down-regulation of miR-517a and miR-517c promotes proliferation of hepatocellular carcinoma cells via targeting Pyk2. Cancer Lett.

[90] Tu Y, Gao X, Li G, Fu H, Cui D, Liu H (2013). MicroRNA-218 inhibits glioma invasion, migration, proliferation, and cancer stem-like cell self-renewal by targeting the polycomb group gene Bmi1. Cancer Res.

[91] Abdelmohsen K, Kim MM, Srikantan S, Mercken EM, Brennan SE, Wilson GM (2010). miR-519 suppresses tumor growth by reducing HuR levels. Cell Cycle.

[92] Pinho FG, Frampton AE, Nunes J, Krell J, Alshaker H (2013). Downregulation of microRNA-515-5p by the estrogen receptor modulates sphingosine kinase 1 and breast cancer cell proliferation. Cancer Res.

[93] Nohata N, Hanazawa T, Enokida H, Seki N (2012). microRNA-1/133a and microRNA-206/133b clusters: dysregulation and functional roles in human cancers. Oncotarget.

